# Prediction of Pasting Properties of Dough from Mixolab Measurements Using Artificial Neuronal Networks

**DOI:** 10.3390/foods8100447

**Published:** 2019-10-01

**Authors:** Georgiana Gabriela Codină, Adriana Dabija, Mircea Oroian

**Affiliations:** Faculty of Food Engineering, Stefan cel Mare University of Suceava, 720229 Suceava, Romania; codina@fia.usv.ro (G.G.C.); adriana.dabija@fia.usv.ro (A.D.)

**Keywords:** white wheat flour, α-amylase, Mixolab, Falling number, artificial neuronal networks

## Abstract

An artificial neuronal network (ANN) system was conducted to predict the Mixolab parameters which described the wheat flour starch-amylase part (torques C3, C4, C5, and the difference between C3-C4and C5-C4, respectively) from physicochemical properties (wet gluten, gluten deformation index, Falling number, moisture content, water absorption) of 10 different refined wheat flours supplemented bydifferent levels of fungal α-amylase addition. All Mixolab parameters analyzed and the Falling number values were reduced with the increased level of α-amylase addition. The ANN results accurately predicted the Mixolab parameters based on wheat flours physicochemical properties and α-amylase addition. ANN analyses showed that moisture content was the most sensitive parameter in influencing Mixolab maximum torque C3 and the difference between torques C3 and C4, while wet gluten was the most sensitive parameter in influencing minimum torque C4 and the difference between torques C5 and C4, and α-amylase level was the most sensitive parameter in predicting maximum torque C5. It is obvious that the Falling number of all the Mixolab characteristics best predicted the difference between torques C3 and C4.

## 1. Introduction

Nowadays, different types of enzymes are used in the bakery field in order to improve the technological processes of the bakery industry and the quality of the baked products [1]. Of these, the most commonly used enzymes are amylases due to their high practical importance. First, amylases provides fermentable sugars for yeast cells and favors the production of carbon dioxide. Second, it contributes to the improvement of dough rheological properties and therefore to the quality of the bakery products [2]. It is well-known that, in general, α-amylase activity is lacking or is present in a very low amount in the wheat flour, whereas the β-amylase is present in a large amount [3,4]. The low level of α-amylase in wheat flour obtained from ungerminated wheat requires further addition due to the fact that β-amylase can only partially hydrolyze starch without the presence of α-amylase [5,6]. Therefore, the importance of adjusting the level of α-amylase activity in wheat flours is essential for bread making. For this purpose, the most common practice used in bread making is the addition of fungal α-amylase during the baking process [7]. Wheat flour with an insufficient amount of α-amylase activity will lead to increased staling rates, thus reducing the shelf life of the finished products and determining a reduced loaf volume and crumb firmness, poor flavor, and pale crust color [1]. However, a high level of α-amylase in wheat flour is also undesirable because it leads to sticky dough, large holes in bread, and gummy crumbs [8].

This explains why the measurement of α-amylase activity in wheat flour is highly recommended. This measurement can be made through both colorimetric and viscometric methods [7]. The colorimetric methods are not usually used by bakery processors due to practical reasons such as price (more expensive per analysis because they involve the use of reagents and require long assay time), low sensitivity, difficulty in continuously monitoring the α-amylase activity in wheat flours in bakery factories, and the fact that they require, personnel with experience in chemical analysis. On the other hand, the viscometric methods are simple, easy to use, not expensive, and compatible with automation.

The most commonly used viscometric methods in practice, which are also the standardized ones, are the Falling number [3,5,9,10,11,12], Brabender amylograph-viscograph [13,14], Rapid Visco Analyser [3,10,11], and the Chopin Mixolab [7,10,12,15]. The Falling number (FN) method is usually used by bakery processors to estimate the α-amylase activity in wheat flour, as the FN is considered one of the most important quality characteristic of wheat flour [5]. The method was developed by Hagberg-Perten in the 1960s and is based on the linear relationship between the α-amylase activity and the liquefaction of the gel starch from wheat flour. The FN value (expressed in second) is associated with wheat flour slurry viscosity, which is negatively correlated with α-amylase activity.The Mixolab device (Chopin Technologies), launched in 2004, was accepted as the ICC standard method No. 173, and has since gained more and more popularity in evaluating the α-amylase activity of wheat flours [7,12,15,16,17]. From all the viscometric methods used to evaluate the α-amylase activity from wheat flour, this is considered the most conclusive for bakery processors due to the fact that it offers valuable information about α-amylase effect during the technological process. In one single test, the Mixolab can make simultaneous determinations of dough rheological properties during mixing and of the changes that occur during the heating process of dough as starch gelatinization and gelling behavior, as well as amylase activity on wheat flour dough [18].

The wheat flour dough is a very complex system affected by different factors and their interactions. For this reason, these factors and interactions must be taken into account when analyzing the dough quality characteristics. In practice, the parameters mostly used to evaluate the wheat flour quality are FN, moisture content, water absorption, and protein quality for bread making, which can be found on any analysis bulletin of wheat flour [19]. Because these parameters are the most common and are critical to assessing the quality of wheat flour, we considered it useful to investigate the prediction of the influence of these parameters alongside different levels of fungal α-amylase addition on pasting properties of wheat flour dough determined with the Mixolab device. For this purpose, we used an artificial neuronal network (ANN): Multilayer perception (MLP), modular neural network (MLP), and probabilistic neural network (PNN). The artificial neural network is a system of information processing that is inspired from the biological nervous system (brain) and is a subfield of computer science. The main advantage of ANN is that it can solve problems without being explicitly programmed and the models can easily reveal the implicit relationship between input and output parameters even if the representation is impossible. This characteristic allows the use of machine learning models in many cases, e.g.,the prediction of different parameters, recognitions, or classifications [20,21,22,23]. The ANN was used for the prediction of different food parameters [22,23,24,25,26].

To our knowledge, no study has been performed on the combined effect of various physicochemical parameters, α-amylase doses, and Falling number values on pasting properties of wheat flour on Mixolab device using an ANN.

## 2. Materials and Methods

### 2.1. Materials

Ten commercial non-additivewhite wheat flour samples of the same type (average value of 0.65 g/100 g ash) analyzed through ICC method 104/2 were purchased from S.C. Mopan S.A. Company (Suceava, Romania). The determination of gluten deformation index, wet gluten content, and moisture content was carried out using the Romanian or international standard methods, as follows: SR 90/2007, ICC standard methods 106/2 and 110/1, respectively. The fungal α-amylase (commercial name Gryndamyl A 14000) derived from a selected strain of *Aspergillus oryzae* produced by Danisco-DuPont (København, Denmark) was added in different levels as 0, 0.02, 0.04 g kg^−1^, 0.06 g kg^−1^ wheat flour. The α-amylase enzyme had an enzymatic activity of 140.000SKB units g^−1^. One SKB unit is the amount of enzyme that will hydrolyze 0.1g of β limit dextrin in one hour. The levels of the α-amylase used were of 0, 2800, 5600, 8400 SKB kg^−1^ wheat flour, which meet the amount of enzyme that dextrinizes (converts to achromic point) 1 g/3-amylase treated starch in one hour at 30°C and pH 4.85.

Ten commercial non-additivewhite wheat flour samples of the same type (average value of 0.65 g/100 g ash) analyzed through ICC method 104/2 were purchased from S.C. Mopan S.A. Company (Suceava, Romania). The determination of gluten deformation index, wet gluten content, and moisture content was carried out using the Romanian or international standard methods, as follows: SR 90/2007, ICC standard methods 106/2 and 110/1, respectively. The fungal α-amylase (commercial name Gryndamyl A 14000) derived from a selected strain of *Aspergillus oryzae* produced by Danisco-DuPont (København, Denmark) was added in different levels as 0, 0.02, 0.04 g kg^−1^, 0.06 g kg^−1^ wheat flour. The α-amylase enzyme had an enzymatic activity of 140.000SKB units g^−1^. One SKB unit is the amount of enzyme that will hydrolyze 0.1g of β limit dextrin in one hour. The levels of the α-amylase used were of 0, 2800, 5600, 8400 SKB kg^−1^ wheat flour, which meet the amount of enzyme that dextrinizes (converts to achromic point) 1 g/3-amylase treated starch in one hour at 30°C and pH 4.85.

### 2.2. Falling Number Analysis

The Falling number (FN) was determined according to ICC standard method 107/1. The method, based on viscosity, was used in order to make a proximate evaluation of the α-amylase activity in wheat flours.

### 2.3. Evaluation of Pasting Properties by Mixolab

The water absorption capacity and the starch pasting properties were determined using Mixolab (Chopin, Tripette et Renaud, Paris, France). The measurements were carried out according to ICC standard method 173. The evaluated parameters were: Water absorption (WA), which corresponds to optimum dough consistency of 1.1 Nm; C3 torque, representing the starch gelatinization; C4 torque, representing the stability of hot starch paste; C5 torque, representing the final starch paste viscosity after cooling at 50°C.The difference between the C3 and C4 peak values (C3-4) corresponds to starch breakdown and the difference between the C5 and C4 peak values (C5-4) corresponds to starch retrogradation at cooling stage.

### 2.4. Data Processing

The software Neurosolution 7.0 trial version (IBM, Florida, USA) was used for the ANN (MLP, PNN, and MNN). The data corresponding to each variable was analyzed by one-factor analysis of variance (ANOVA) and linear regression using Statgraphics XVII trial version (Statgraphics Technologies, Inc., USA). Multiple comparisons were performed using the least significant difference test (LSD) and Fisher ratio (F), and statistical significance was set at α = 0.05.

The prediction of the rheological parameters (C3, C4, C5, C3-C4, and C5-C4—output parameters) was done in function of the input parameters (wet gluten, gluten deformation index, water absorption, Falling number, moisture content, and α-amylase level). The model used for the prediction of the rheological parameters were: MLP, MNP, and PNN. Each model was applied using a different number of hidden layers (intermediate layer between the input and output layer) ranging from one to three. For achieving the suitable model, some statistical parameters were checked: Mean squared error (MSE), regression coefficients (R^2^), and mean absolute error (MAE). The input experimental data (40 samples) was divided into three categories: Training (50% of the experimental data), cross validation (25% of the experimental data), and testing (25% of the experimental data). The sensitivity analysis (%) investigated the effect of each input parameter on the output in terms of magnitude and direction.

## 3. Results and Discussion

### 3.1. Analytical Characteristics of Wheat Flours

The content of wet gluten, gluten deformation index, water absorption, Falling number, and moisture content of the refined wheat flours of 650 type are shown in Table 1. The quality of the used wheat flours covers a wide range of breadmaking qualities. The samples used in the analysis presented high FN values (>330 s) which, according to Ji, Penning, and Baik, indicates low α-amylase activity [9].

### 3.2. Influence of Fungal α-Amylase Addition on the Falling Number Values

**Figure 1:**
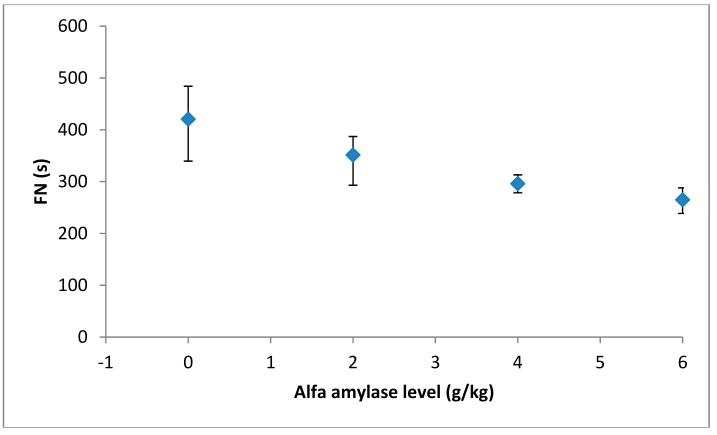
Of fungal α-amylase addition on Falling number values.

Different levels of fungal α-amylase were added to wheat flour (0, 0.02, 0.04 g kg^−1^) and the Falling number values of the resulting dough samples were measured. The graphical representation of the FN values in relation to the level of *α*-amylase addition is shown in Figure 1. It can be seen that with the increased level of α-amylase addition, the FN decreased up to levels corresponding to optimum FN values (250–300 s). Therefore, the quality of wheat flours was improved up to a normal α-amylase activity and, from this point of view, the flours were acceptable for breadmaking [5]. The significant decrease of the Falling number value with the increase level of amylase addition is due to the fungal amylase type which is specially formulated for breadmaking, and, according to enzyme producer, is quite stable and improves with both the baking performance of the flour and the Falling number value.

### 3.3. Influence of Fungal α-Amylase Addition on the Mixolab Pasting Properties

Wheat flour dough rheological properties related to starch pasting properties and amylase activity were analyzed with the Mixolab device. As can be seen in Table 2, the α-amylase addition in wheat flour dough decreased all the Mixolab parameters values, having a significant effect (*p* < 0.001) on torques C3, C4, C5, and the difference between torques C5 and C4. This result can be attributed to the fact that dough temperature increases up to values that favor starch gelatinization and intensifies the α-amylase activity. Partially gelatinized starch is more easily hydrolyzed by amylases than the raw starch. A higher starch hydrolysis leads to an increase of the amount of simple sugars and low molecular weight dextrin in dough system which causes a decreased in dough consistency [27]. Therefore, with the increase level of α-amylase activity, there will be a lager difference between the C3, C4, and C5 Mixolab parameters. Similar results were also reported [7,15,18].

### 3.4. Mixolab Pasting Parameters Prediction

The prediction of Mixolab parameters was made using two techniques—linear regression and artificial neural networks—in order to achieve the suitable method for the prediction.

#### 3.4.1. Mixolab Pasting Parameters Prediction Using Linear Regression

Table 3 presents the equations and regression coefficients of Mixolab pasting parameters prediction using linear regression. As it can be observed, the regression coefficients of the equations are lower than 0.7, and in the case of C3-C4, the coefficient is lower than 0.3.

#### 3.4.2. Mixolab Pasting Parameters Prediction Using ANN

Table 4 presents the statistical parameters for each model analyzed. It can be observed that for C4 (R^2^ ≥ 0.893, MAE ≤ 0.069, MSE ≤ 0.008), C5 (R^2^ ≥ 0.946, MAE ≤ 0.086, MSE ≤ 0.012), and C5-C4 (R^2^ ≥ 0.907, MAE ≤ 0.096, MSE ≤ 0.014), the suitable model is the PNN with two hidden layers; for C3 (R^2^ ≥ 0.893, MAE ≤ 0.069, MSE ≤ 0.008), the suitable model is MNN with one hidden layer; and for C3-C4 (R^2^ ≥ 0.8880, MAE ≤ 0.090, MSE ≤ 0.013), the suitable model is MNN with two hidden layers, respectively. It can be observed that increasing the number of hidden layers did not cause the regression coefficients of the models to increase.

**Figure 2:**
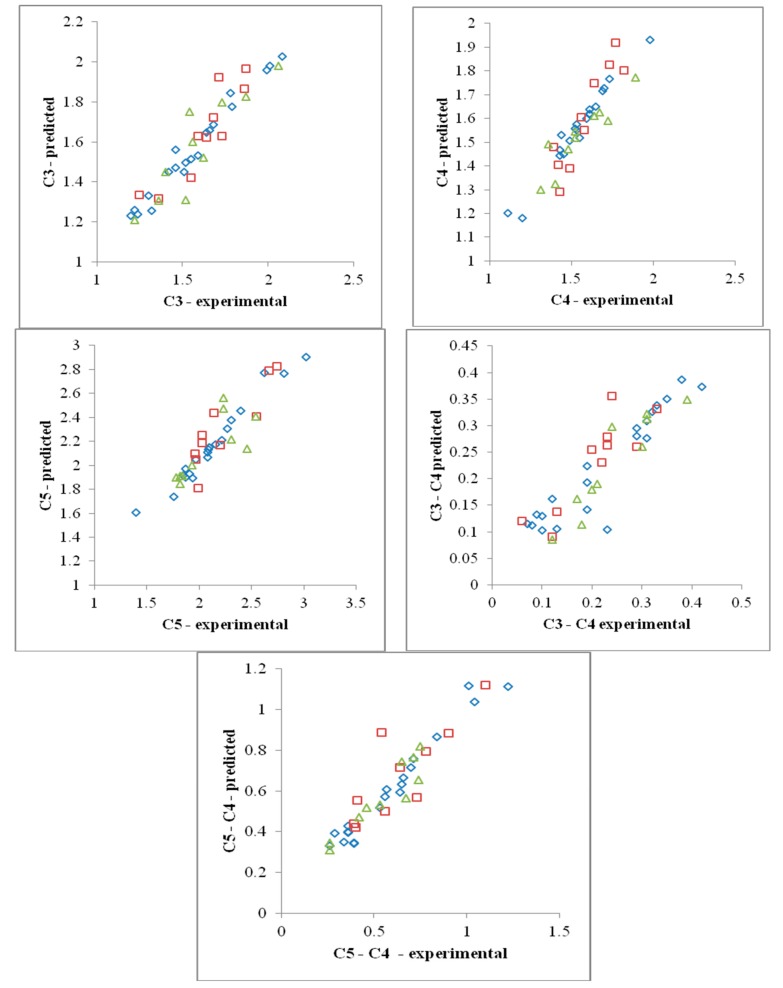
Experimental vs. predicted data using an artificial neural network (ANN) for dough rheological parameters: C3 (MNN—one hidden layer), C4 (PNN—two hidden layers), C5 (PNN—two hidden layers), C3-C4 (MNN—two hidden layers), and C5-C4 (PNN—two hidden layers), rhombus—training, triangle—cross validation, square—testing.

Figure 2 presents the evolution of the experimental versus predicted data for the C3, C4, C5, C3-C4, and C5-C4 parameters in function of wet gluten, gluten deformation index, water absorption, Falling number, moisture content, and α-amylase levels by the best model for each output parameter using the artificial neural network (according to the data in Table 5).

A sensitivity analysis was performed to investigate the effect of each input parameter on the output in terms of magnitude and direction (Table 5). The sensitivity analysis is a simple and powerful tool to evaluate a system’s behavior. With wide applications in science and engineering, it is a critical step in the mathematical modelling of the hydrometeorological processes. A large sensitivity to a parameter suggests that the system’s performance can drastically change with a small variation in the parameter [28]. Moisture content was the most sensitive for C3 and C3-C4, while wet gluten was the most sensitive for C4 and C5-C4, and α-amylase levels were the most sensitive for C5, respectively. The maximum peak C3 value, corresponding to starch gelatinization, was the most affected by moisture content and water absorption values of wheat flour samples.

This effect is most likely correlated with the way in which the samples were prepared. Mixolab software calculates the amount of flour that can be introduced in the device tank and the amount of water that is required to be injected in order to obtain the standard consistence of wheat flour dough of 1.1 Nm according to the moisture content value of the flour sample. Also, all the samples in which α-amylase was incorporated were prepared at the same water absorption value, according to the input value of flour moisture, at constant hydration. It is well-known that α-amylase addition leads to an increase in the amount of maltose in the dough, which decreases the water absorption of the wheat flour [2,29]. Using a constant hydration for all the wheat flour samples, the amount of free water in the dough in which α-amylase was incorporated will increase, leading to an increase of the mobility of the α-amylase enzyme and substrate molecules. The intensification of the amylolytic activity will lead to a higher degradation of the damaged starch which will absorb water in a higher amount that the undamaged starch [1]. This fact, alongside the increased water content from the dough system, will make the starch gelatinization more complete. From Table 5, it can be seen that after moisture content and water absorption of the wheat flour used in analysis, two parameters that showed sensitivity on Mixolab maximum peak C3 value were the FN values and the amylases doses. This might be explained by the fact that FN is a value measured based on the viscosity of the wheat flour slurry heated up to 100°C and is linked to the α-amylase activity and starch behavior during the gelatinization process. The FN value is inversely proportionate to the wheat flour slurry viscosity, being an indirect measurement of α-amylase activity and the level of damaged starch from the wheat flour [10]. At the Mixolab device, the maximum peak C3 value will increase with the increase of the starch gelatinization capacity and the decrease of the α-amylase activity. Therefore, in general, it can be seen that the most sensitive parameters on C3 peak are moisture content and water absorption, followed by Falling number and α-amylase doses. At the end of the starch gelatinization stage, the Mixolab device reached the optimal temperature for the α-amylase activity, but its activity on wheat flour dough was short.

The difference between the C3 and C4 peak values (Nm) corresponds to the starch breakdown and expresses the rate of enzymatic hydrolysis on wheat flour starch. The dough temperature increased from 60 °C to 90 °C, and this change gradually decreased the α-amylase activity on wheat flour starch. The factors mentioned before (moisture content and water absorption) that influence the amylase hydrolysis activity on dough system, alongside FN value, which is a standard parameter that measures the α-amylase activity and α-amylase doses added in wheat flour, had the greatest influence on the C3-4 difference. It can be seen that the same parameters of the wheat flour sample, in the same order as in the case of the C3 value, presented the highest sensitivity on this parameter. Similar results were also obtained by [30,31]. In [31], it was reported that from all the Mixolab data, the C3-4 difference presented the highest correlation with FN.

The C4 torque value measures the stability of hot starch paste. The dough temperature is within the limits of the α-amylase activity and, therefore, its period of action on the gelatinized starch is quite high. The presence of the fungal α-amylase, which also contains low amounts of proteolytic enzymes, results in the formation of maltose, which exerts a dehydration action on gluten, thus increasing the liquid phase of the dough. Consequently, the dough consistency expressed through C4 torque will decrease. As we expected, the amylase doses predicted the C4 torque values. After the wet gluten content of wheat flours, this was the second experimental parameter with high sensitivity on this parameter. In wheat flours richer in proteins and therefore in wet gluten, the starch content decreased [32]. The reduced amount of starch from wheat flour, due to its higher protein content, may contribute to a decrease inthe C4 values, which indicate starch gel stability. Also, this may lead to a lower amount of gelatinized starch, thus affecting the α-amylase activity which will have a lower substrate to act on. Moreover, wet gluten is the main parameter that determines the dough rheological properties, being directly involved in the protein-starch interactions through hydrogen bonding and directly responsible for the semi-rigid structure of baked products [27]. According to Eliasson (1983), it seems that the interactions between wet gluten and starch during the heating process decrease the viscosity values, which are lower in the presence of gluten proteins compared to the ones determined when only the pure starch is involved [33]. Wheat flour dough properties are affected by the changes of gluten network during heating, which seems to induce a re-aggregation of protein molecules with an increase of gluten elasticity over 55 °C, leading to a higher dough thermal stability [34]. The significant influence of the amount of gluten proteins on hot starch paste of wheat flour dough of the Mixolab device were reported in [35,36,37].

The C5-4, expressing the retrogradation rate of the wheat flour dough samples, is also mostly influenced by the wet gluten content parameter. These results show that wheat flour dough rich in gluten is able to form a strong gel after heating during the cooling phase. It was reported that the increase of the amount of gluten content decreased the staling rate of bread and that it may present an anti-firming effect on starch [38]. However, Xijun et al. (2014) reported that gliadins, one of the gluten proteins, significantly increased the retrogradation rate of wheat starch, being one of the α-amylase inhibitors [39]. They reported that the amount of water available to the starch is altered by gluten, which may slow down the motion of starch chains favoring their aggregation and thus increase the starch retrogradation process. References [40,41] reported that, from the wheat flour components, gluten has the main role in staling process as a result of hydrogen bonding between gluten network and gelatinized starch granules, which may lead to a continuous protein network and discontinuous granule remnants [24]. Gerrard et al. completed the hypothesis previously mentioned before concluding that the staling rate of bread with α-amylase addition is a direct result of the interaction between the starch granules and proteins, which are reduced due to starch hydrolysis products not specifically associated with the protein network [42].

## 4. Conclusions

The α-amylase addition significantly influenced (*p* < 0.01) almost all the Mixolab parameters evaluated (torques C3, C4, C5, and the difference between torques C5 and C4), which displayed decreased values. According to ANN system, the Mixolab parameters values are sensitively influenced by the physicochemical properties of wheat flours as follows: C3 and the difference between torques C3 and C4 by moisture content and water absorption, and C4 and the difference between torques C5 and C4 by wet gluten content of wheat flours. The α-amylase addition level in wheat flour mostly influenced the Mixolab C5 torque corresponding to the final starch paste viscosity.

## Figures and Tables

**Table 1:** Characteristics of wheat flour.

Parameters	Mean (min–max)	F-Value
**Wet gluten (%)**	27.1 (24.3–30.0)	14.01 ***
**Gluten deformation index (mm)**	6.8 (3.9–14.0)	764.8 ***
**Water absorption (%)**	57.2 (54.6–59.4)	2.25ns
**Falling number (s)**	420.8 (339.5–484.0)	39.56 ***
**Moisture content (%)**	14.4 (13.8–15.1)	2.71ns

*ns-not significant* (*p* > 0.05), ***- *p* < 0.001.

**Table 2:** Of α-amylase addition on Mixolab pasting properties.

Parameters	α-Amylase Level (g kg^−1^)				F-Value
	0	2	4	6	
**C3 (Nm)**	1.77a	1.60b	1.48bc	1.4 °C	13.69 ***
**C4 (Nm)**	1.67a	1.54b	1.47bc	1.43c	10.30 ***
**C5 (Nm)**	2.49a	2.11b	2.00b	1.85c	28.76 ***
**C3-C4 (Nm)**	0.22	0.24	0.23	0.19	0.88^ns^
**C5-C4 (Nm)**	0.82a	0.58b	0.53bc	0.42c	16.44 ***

C3, maximum torque-measure of starch gelatinization; C4, minimum torque-measure of stability of hot starch paste; C5, maximum torque-measure of the final starch paste viscosity; C3-4, difference between torques C3 and C4;C5-4, deference between torques C5 and C4. *ns-not significant* (*p* > 0.05), ***-*p* < 0.001.

**Table 3:** Linear regression equation and regression coefficients of Mixolab pasting parameters prediction.

Parameter	Equation	R^2^
C3	C3 = 4.542 + 0.001·A − 0.027·B-0.042·C + 0.001·D − 0.074·E	0.600
C4	C4 = 0.938-0.092·A-0.05·B + 0.052·C + 0.001·D − 0.025·E	0.683
C5	C5 = 5.506 + 0.372·A + 0.117·B + 0.005·C + 0.001·D + 0.001·E	0.651
C3-C4	C3-C4 = 0.292 + 0.042·A + 0.001·B − 0.027·C + 0.001·D + 0.016·E	0.267
C5-C4	C5-C4 = 2.746 + 0.065·A-0.015·B-0.084·C + 0.003·D + 0.012·E	0.552

A—wet gluten, B—gluten deformation index, C—water absorption, D—Falling number, E—moisture content.

**Table 4:** Statistical parameters for rheological parameters of dough.

No	Model Name ^1^	Hidden Layers	Training			Cross Validation			Testing		
			MSE	R^2^	MAE	MSE	R^2^	MAE	MSE	R^2^	MAE
**C3**											
1	MLP	1	0.001	0.986	0.031	0.014	0.845	0.107	0.020	0.802	0.109
2	MLP	2	0.002	0.976	0.043	0.015	0.871	0.102	0.027	0.720	0.122
3	MLP	3	0.006	0.964	0.069	0.023	0.780	0.122	0.014	0.871	0.101
4	PNN	1	0.001	0.990	0.028	0.016	0.863	0.110	0.016	0.863	0.110
5	PNN	2	0.002	0.983	0.045	0.017	0.807	0.111	0.012	0.897	0.093
6	PNN	3	0.006	0.959	0.069	0.008	0.901	0.081	0.010	0.904	0.088
7	MNN	1	0.001	0.986	0.032	0.009	0.910	0.078	0.013	0.880	0.090
8	MNN	2	0.001	0.986	0.036	0.020	0.820	0.114	0.012	0.899	0.092
9	MNN	3	0.006	0.970	0.070	0.008	0.882	0.078	0.008	0.930	0.083
**C4**											
1	MLP	1	0.001	0.984	0.0244	0.005	0.861	0.063	0.006	0.892	0.064
2	MLP	2	0.001	0.988	0.0288	0.014	0.845	0.097	0.011	0.857	0.085
3	MLP	3	0.008	0.886	0.068	0.007	0.801	0.065	0.006	0.941	0.106
4	PNN	1	0.001	0.971	0.035	0.004	0.910	0.051	0.004	0.910	0.051
5	PNN	2	0.001	0.983	0.028	0.008	0.893	0.069	0.006	0.931	0.073
6	PNN	3	0.009	0.855	0.066	0.011	0.746	0.081	0.013	0.751	0.095
7	MNN	1	0.005	0.917	0.058	0.004	0.918	0.051	0.005	0.921	0.057
8	MNN	2	0.004	0.923	0.048	0.010	0.899	0.077	0.009	0.879	0.072
9	MNN	3	0.022	0.555	0.120	0.007	0.805	0.077	0.018	0.615	0.117
**C5**											
1	MLP	1	0.004	0.982	0.043	0.039	0.735	0.174	0.026	0.873	0.140
2	MLP	2	0.008	0.968	0.067	0.054	0.686	0.181	0.036	0.734	0.141
3	MLP	3	0.010	0.965	0.080	0.065	0.658	0.171	0.034	0.855	0.170
4	PNN	1	0.005	0.979	0.049	0.026	0.835	0.125	0.026	0.835	0.125
5	PNN	2	0.006	0.980	0.061	0.012	0.946	0.084	0.010	0.951	0.086
6	PNN	3	0.024	0.905	0.112	0.018	0.899	0.114	0.041	0.864	0.172
7	MNN	1	0.009	0.966	0.071	0.007	0.956	0.072	0.021	0.876	0.127
8	MNN	2	0.009	0.963	0.075	0.012	0.957	0.096	0.025	0.846	0.147
9	MNN	3	0.034	0.873	0.132	0.026	0.827	0.126	0.025	0.819	0.121
**C3-C4**											
1	MLP	1	0.004	0.982	0.043	0.003	0.877	0.048	0.004	0.667	0.052
2	MLP	2	0.001	0.940	0.027	0.003	0.808	0.043	0.005	0.714	0.058
3	MLP	3	0.001	0.928	0.030	0.004	0.870	0.054	0.003	0.756	0.042
4	PNN	1	0.001	0.983	0.016	0.011	0.479	0.086	0.011	0.479	0.086
5	PNN	2	0.001	0.996	0.007	0.007	0.518	0.070	0.005	0.725	0.049
6	PNN	3	0.001	0.954	0.029	0.007	0.310	0.080	0.007	0.317	0.069
7	MNN	1	0.001	0.929	0.036	0.005	0.797	0.063	0.005	0.594	0.055
8	MNN	2	0.001	0.969	0.021	0.004	0.812	0.048	0.003	0.837	0.040
9	MNN	3	0.008	0.609	0.084	0.005	0.333	0.067	0.005	0.330	0.059
**C5-C4**											
1	MLP	1	0.002	0.981	0.037	0.027	0.720	0.130	0.018	0.870	0.116
2	MLP	2	0.007	0.945	0.071	0.034	0.644	0.146	0.021	0.716	0.124
3	MLP	3	0.003	0.981	0.043	0.056	0.538	0.161	0.040	0.694	0.165
4	PNN	1	0.001	0.989	0.029	0.026	0.735	0.121	0.026	0.735	0.121
5	PNN	2	0.003	0.973	0.052	0.006	0.941	0.063	0.014	0.907	0.096
6	PNN	3	0.014	0.895	0.092	0.029	0.712	0.080	0.012	0.863	0.085
7	MNN	1	0.002	0.979	0.043	0.012	0.861	0.097	0.011	0.880	0.091
8	MNN	2	0.004	0.970	0.057	0.013	0.850	0.100	0.012	0.875	0.102
9	MNN	3	0.021	0.854	0.121	0.026	0.679	0.112	0.014	0.817	0.098

^1^ MLP—multilayer perceptron, PNN—probabilistic neural network, MNN- modular neural network.

**Table 5:** Testing (%) of wet gluten, gluten deformation index, water absorption, Falling number, moisture content, and α-amylase levels (ANN) for predicting the rheological parameters (C3, C4, C5, C3-C4, and C5-C4): PNN—two hidden layers for C3, C4, and C5-C4; MNN—one hidden layer for C3; and MNN—two hidden layers for C3-C4.

Parameter Sensitivity	C3	C4	C5	C3-C4	C5-C4
**Wet gluten**	5.23	32.07	13.48	7.27	28.77
**Gluten deformation index**	5.48	12.73	9.02	5.44	9.30
**Water absorption**	24.23	16.77	16.68	24.26	9.89
**Falling number**	22.29	6.81	13.04	13.99	9.10
**Moisture content**	30.63	11.84	18.20	35.99	22.59
**α-amylase levels**	12.11	19.75	29.56	13.02	20.33
